# Importance of OsRac1 in Signalling of Pigm-1 Mediated Resistance to Rice Blast Disease

**DOI:** 10.3390/plants14020217

**Published:** 2025-01-14

**Authors:** Dewei Yang, Niqing He, Fenghuang Huang, Jialin Chen, Minxiang Yu, Yidan Jin, Shaojun Lin, Shengping Li

**Affiliations:** 1 Institute of Rice, Fujian Academy of Agricultural Sciences, Fuzhou 350018, China; heniqing430@163.com (N.H.); hhf13305022536@163.com (F.H.); mxyu0037@163.com (M.Y.); shaojunlin1993@163.com (S.L.); 2 College of Agriculture and Plant Immunity Center, Fujian Agriculture and Forestry University, Fuzhou 350002, China; jialinchen001@163.com (J.C.); stml093015@163.com (Y.J.); 3 Fujian Provincial Key Laboratory of Crop Breeding by Design, Fujian Agriculture and Forestry University, Fuzhou 350002, China

**Keywords:** Pigm-1, OsRac1, rice blast resistance, signal transduction, molecular breeding

## Abstract

In rice, leucine-rich repeat nucleotide-binding site (NLR) proteins are pivotal immune receptors in combating *Magnaporthe oryzae*-triggered rice blast. However, the precise molecular mechanism underlying how NLR proteins regulate downstream signalling remains elusive due to the lack of knowledge regarding their direct downstream targets. The NLR protein Pigm-1 was cloned from Shuangkang 77009 in our laboratory. This study shows that the nucleotide-binding site (NBS) domain of Pigm-1 facilitates its binding to and activation of OsRac1 while the coiled-coil (CC) domain enables its binding to and activation of RAI1, ultimately inducing cell death. At the same time, after knocking out *OsRac1* in the background of Shuangkang 77009 containing *Pigm-1*, two knockout lines showed susceptibility to rice blast. This study reveals OsRac1, a GTPase, as a signalling molecule involved in Pigm-1-mediated blast resistance, suggesting its potential as a common downstream effector of rice NLR proteins. Additionally, a transcriptional activator, RAI1, acts as an essential Pigm-1 interactor for blast resistance. Furthermore, a novel material 9311(*Pigm-1*) was prepared by using two-line restorer line 9311 as receptor and Shuangkang 77009 as donor with molecular marker-assisted technology, which improved blast resistance and yield. This research demonstrates that molecular marker-assisted selection technology enhances both resistance and yield in the crucial two-line restorer 9311(*Pigm-1*). This study offers crucial insights into how Pigm-1 protein activates downstream molecules and serves as a valuable reference for the molecular breeding of rice blast resistance genes, particularly *Pigm-1*.

## 1. Introduction

The plant innate immune system can be divided into two layers, depending on the immune receptors. One layer involves a plasma membrane (PM)-anchored pattern recognition receptor, which senses conserved pathogen structural molecules called pathogen-associated molecular patterns to induce a basic form of resistance called pathogen-associated molecular pattern triggering immunity (PTI). The other layer relies on resistant (R) proteins, most of which belong to the nucleotide-binding site leucine-rich repeat sequence (NLR) protein family. NLR proteins contain a central nucleotide-binding site (NBS), a C-terminal leucine-rich repeat (LRR) sequence, and either a variable N-terminal coiled-coil (CC) domain or a Toll/interleukin-1 receptor (TIR) domain. Plant R proteins that recognise pathogen effector proteins lead to pathogen race-specific resistance, known as effector-triggered immunity (ETI) [1,2,3]. The immune responses induced by ETI are generally more rapid, prolonged, and robust than those induced by PTI [4,5]. Recent studies have shown that PTI and ETI are closely related and enhance each other, providing strong disease resistance. Plant PTI is an indispensable part of ETI in the process of pathogen infection, and in turn, the activation of ETI can enhance the immune response stimulated by PTI [6,7].

In addition, R proteins are intracellular receptors that recognise strain-specific effectors (also known as avirulence proteins, AVRs) to trigger rapid and enhanced immune responses, including defence-related genes, bursts of reactive oxygen species (ROS), sustained increases of cytoplasmic Ca^2+^, and even locally programmed cell death, known as hypersensitivity [8]. *R* gene-induced defences are usually very resistant to invaders, so plant *R* genes are an important genetic resource for crop breeding [9,10].

Members of the small GTPase Rac/Rop family act as molecular switches and play crucial roles in a variety of plant physiological processes [11,12]. OsRac1 is a key signal switch downstream of two types of immune receptors, pattern recognition receptor and resistance protein, and can trigger innate immunity [13,14,15]. Research has revealed that an OsCERK1–OsRacGEF1–OsRac1 module is involved in early signalling for chitin-induced immunity [16]. A chitin receptor complex containing RLP OsCEBiP and RLK OsCERK1 was found to phosphorylate OsRacGEF1, the PRONE family activating protein of OsRac1. The OsCERK1-dependent phosphorylation of OsRacGEF1 results in the activation of OsRac1, leading to the induction of an immune response. Hsp90 and its common partner Hop/Sti1 complex were found to contribute to the maturation and intracellular transport of the OsCERK1 complex [17].

It has been reported that OsRac1 can interact with two NLR proteins, Pit and Pia, when pathogens invade rice, thus playing an important role in the immune response mediated by these NLR proteins [18,19]. Our recent studies have found that OsRac1 is a signalling molecule of rice blast resistance mediated by Pid3 and Pi9 and may be a common factor downstream of the rice NLR protein [20]. However, for R proteins other than Pit, Pia, Pi9, and Pid3, it is unclear whether the activation of blast resistance is equally conserved in the OsRac1 signalling pathway. Therefore, exploring whether the OsRac1 signalling pathway has a similar function in a broader range of R proteins has important implications for enhancing our understanding of the conservation nature of this pathway.

Previously, we cloned *Pigm-1*, a new allele of the broad-spectrum rice blast resistance gene *PigmR*, from Shuangkang 77009 by mapping cloning [21]. In this study, we found that Pigm-1 associated with OsRac1 through its NBS domain and regulated OsRac1 activation while OsRac1 is important for blast resistance mediated by Pigm-1. Our results provide more mechanistic clues about how different types of NLR proteins trigger the activation of downstream molecules.

## 2. Results

### 2.1. Amino Acid Sequence Analysis and Comparison Between Pigm-1 and Pit, Pia, Pid3, Pi9

It has been confirmed that the Pit, Pia, Pid3, and Pi9 NLR proteins interact with OsRac1 and that OsRac1 plays an important role in the immune response mediated by these three NLR proteins, so OsRac1 is likely to be a common downstream factor [18,19,20]. To further analyse the homology between Pigm-1 and the four R proteins Pit, Pia, Pid3, and Pi9, we compared the amino acid sequences of these five R proteins. The results indicated that Pigm-1 was a distant homologue of Pit, Pia, and Pid3 (Supplementary Figure S1a) and a close homologue of Pi9 (Supplementary Figure S1b).

### 2.2. Pigm-1 Interacts with OsRac1 by the NBS Domain

**Figure 1:**
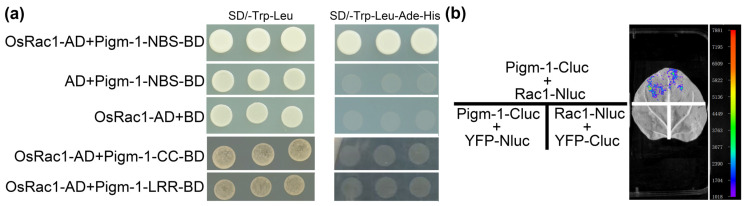
Pigm-1-NBS interacts with OsRac1. (**a**) The interaction between Pigm-1-NBS and OsRac1 was detected by the Y2H assay. The corresponding vectors were cotransformed into yeast, and the transformed yeast was cultured on the defective medium (SD/-Trp-Leu, SD/-Trp-Leu-Ade-His). Each combination was repeated three times. (**b**) Pigm-1 interacted with OsRac1 in the firefly split-luciferase complementation (LUC) assay. The indicated constructs were transiently coexpressed in 4-week-old *N. benthamiana* leaves. Cluc and Nluc empty vectors were used as negative controls.

To investigate which domain of Pigm-1 is responsible for its interaction with OsRac1, we generated several truncated forms of Pigm-1 protein, the LRR, the NBS, and the CC, and performed a yeast two-hybrid (Y2H) assay. The results demonstrated that the NBS of Pigm-1 interacted with OsRac1 (Figure 1a) while the LRR and CC of Pigm-1 did not interact with OsRac1 (Figure 1a). Therefore, these experiments indicated that the NBS domains of Pigm-1 are necessary and sufficient for their physical interaction.

To further verify their interaction, we performed a LUC assay in *N. benthamiana* leaves. As shown in Figure 1b, luminescent signals were detected in leaves when OsRac1-Nluc and Pigm-1-Cluc were coexpressed but not in the negative controls.

### 2.3. Amino Acid Sequence Analysis of OsRac1 in Different Rice Varieties

**Figure 2:**
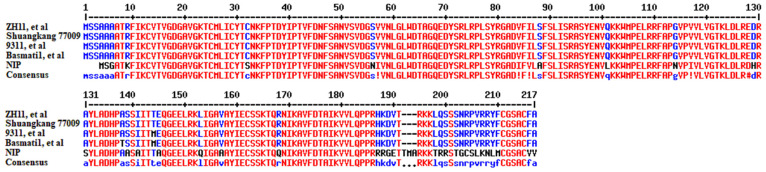
Amino acid sequence analysis of OsRac1 in 33 sequenced rice varieties. ZH11, CG14, KY131, LJ, NamRoo, Kosh, and DHX2 showed the same results as OsRac1; 9311, 02428, IR64, CN1, D62, R498, Lemont FH838, FS32, G46, G8, G630, II32, J4155, DG, Y3551, R527, S548, Tumba, WSSM, Y58S and YX1 showed one amino acid difference (T144 changed to M144); Basmati1, TM, and N22 showed two different amino acid changes (T144 to M144 and A138 to T138); NIP showed the presence of many individual amino acid variations; and Shuangkang 77009 sequencing revealed the same results as OsRac1.

To further analyse the amino acid sequence changes in OsRac1 in different rice materials, we found four different types of OsRac1 changes based on 33 rice varieties that have completed three-generation deep sequencing (https://ricerc.sicau.edu.cn/). The first type of material comprises the ZH11, CG14, KY131, LJ, NamRoo, Kosh, and DHX2 varieties, and their sequences are exactly the same as that of OsRac1. The second type has one amino acid difference (T144 changed to M144) in the 9311, 02428, IR64, CN1, D62, R498, Lemont, FH838, FS32, G46, G8, G630, II32, J4155, DG, Y3551, R527, S548, Tumba, WSSM, Y58S, and YX1 varieties. The third type, comprising the Basmati1, TM, and N22 varieties, had two different amino acid changes (T144 to M144 and A138 to T138). The fourth type, represented only by the NIP variety, contains many individual amino acid variations (Figure 2 and Supplementary Table S1).

To determine the *OsRac1* sequence in Shuangkang 77009, we sequenced the gene (sequencing primers are shown in Supplementary Table S2) and performed a translational comparison of the sequencing results. The results showed that the amino acid sequence of OsRac1 in Shuangkang 77009 was the same as that of the wild-type OsRac1 (Figure 2).

### 2.4. OsRac1 Is a Key Downstream Component of the Pigm-1 Protein

**Figure 3:**
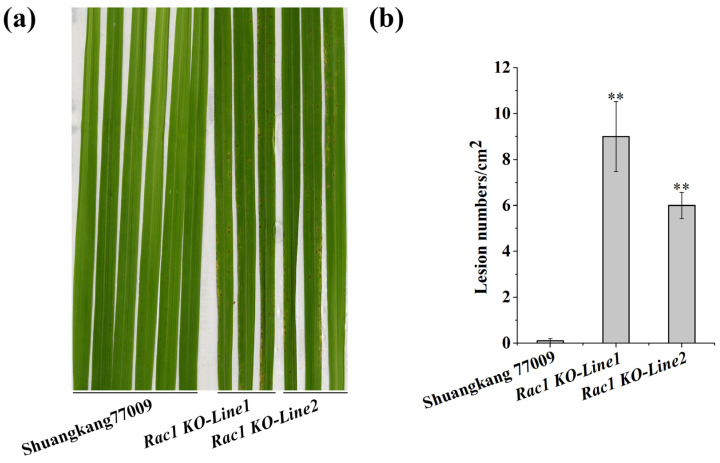
*OsRac1* knockout mutants in the Shuangkang 77009 background show enhanced susceptibility to Guy11 compared to Shuangkang 77009. (**a**) The inoculation of rice blast fungus assay showed that two knockout lines generated by CRISPR/Cas9 were susceptible to Guy11, while Shuangkang 77009 was resistant to Guy11. The leaves were photographed 7 days post-infection with *M. oryzae* isolate Guy11. (**b**) Lesion numbers per cm^2^ on the rice leaves (M ± SE) in (**a**). Asterisks indicate statistical significance determined by Dunnett’s *t* test. **, *p* ≤ 0.01.

To further determine the role of *OsRac1* in disease resistance, we knocked out the *OsRac1* gene in the background of Shuangkang 77009. We obtained a total of two homozygotes from two independent knockout events and confirmed the presence of insertion and deletion mutations at the target sites by Sanger DNA sequencing (Supplementary Table S2), and the two lines were named *Rac1 KO-Line1* and *Rac1 KO-Line2*, respectively. We then inoculated the two knockout lines with Guy11. The results showed that Shuangkang 77009 exhibited almost no susceptibility to the disease while both *Rac1 KO-Line1* and *Rac1 KO-Line2* had rice blast lesions on their leaves (Figure 3a). Compared to the lesion numbers of Shuangkang 77009, the lesion numbers on the leaves of *Rac1 KO-Line1* and *Rac1 KO-Line2* reached to significant levels (Figure 3b). In addition, analysis of main agronomic traits showed that there were no significant differences in plant height, panicle length, effective panicle number, number of grains per panicle, seed-setting rate, 1000-grain weight, grain length, or grain width between Shuangkang 77009 and the two knockout transgenic lines (Supplementary Table S3). We speculated that *OsRac1* mainly plays a role in rice disease resistance but does not affect other agronomic traits.

The *Pigm-1* gene contributes blast resistance to Shuangkang 77009. However, after *OsRac1* was knocked out in the background of Shuangkang 77009, the two knockout lines showed susceptible phenotypes. These results indicated that OsRac1 plays an important role in plant disease resistance and is a key downstream component of the Pigm-1 protein.

### 2.5. Pigm-1 Is Used to Improve Blast Resistance in 9311

In a previous study, *Pigm-1*, a broad-spectrum rice blast resistance gene, was cloned from Shuangkang 77009 by mapping cloning [21]. To improve the resistance of 9311 to rice blast, we used 9311 (Food Crops Research Institute, Jiangsu Academy of Agricultural Sciences, Nanjing, China), an *indica* cultivar, as a recipient and Shuangkang 77009 (Rice Research Institute, Fujian Academy of Agricultural Sciences, Fuzhou, China), a restorer *indica* cultivar, as a donor. F_1_ plants were generated from 9311 as the female parent and Shuangkang 77009 as the male parent. The F_1_ plants were backcrossed to the 9311 parent to produce the BC_1_F_1_ generation. These BC_1_F_1_ plants were backcrossed to the 9311 parent to produce BC_2_F_1_ plants. In the same way, BC_3_F_1_ and BC_4_F_1_ individuals were obtained. Marker-assisted selection (MAS) with *Pigm-1* was conducted on 22 BC_4_F_2_ lines, which were selected at random by taking three from each line, and one of the lines was designated 9311(*Pigm-1*) (Supplementary Figure S2).

**Figure 4:**
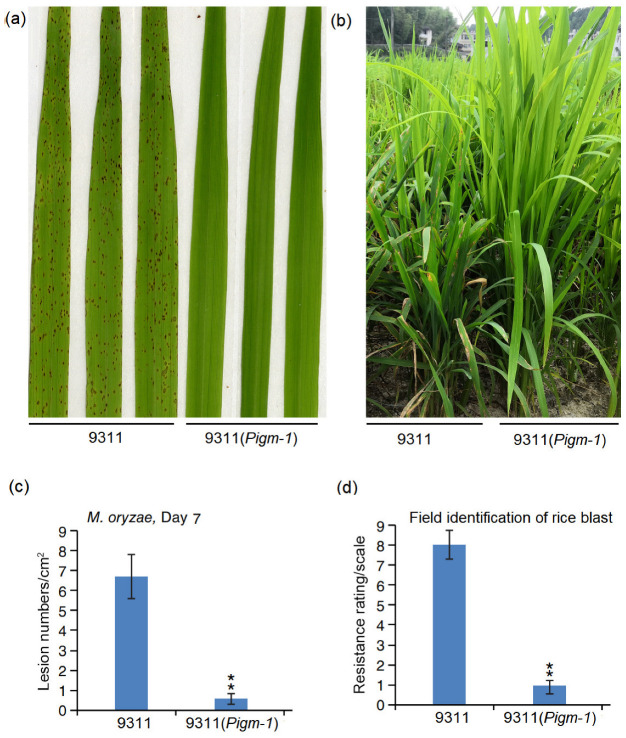
The disease symptoms of the rice cultivars 9311 and 9311(*Pigm-1*) infected by blast fungi. (**a**) Blast resistance of 9311 and 9311(*Pigm-1*) plants using spraying inoculation in a greenhouse. Representative leaves obtained 7 days after inoculation with blast strain Guy11. (**c**) Lesion numbers per cm^2^ on the rice leaves (M ± SD, n > 10 leaves) in (**a**). Asterisks indicate statistical significance (*p* < 0.01) determined by Student’s *t* test. (**b**) 9311(*Pigm-1*) exhibited strong leaf blast resistance in a natural nursery in Shanghang, Fujian Province, China. (**d**) The disease score of 9311 is 8, and that of 9311(*Pigm-1*) is 1.

To analyse the blast resistance of 9311(*Pigm-1*) under laboratory conditions, a rice blast inoculation experiment was performed with the *M. oryzae* strain Guy11. With the susceptible rice cultivar 9311 as a control, we observed the development of blast lesions on the leaves of 9311, whereas 9311(*Pigm-1*) exhibited resistance to rice blast fungus Guy11 (Figure 4a). The number of lesions on the leaves of 9311(*Pigm-1*) was significantly lower than that of 9311 (Figure 4c). However, experimental results under laboratory conditions are often influenced by various factors, necessitating further verification under natural field conditions. Therefore, we planted 9311(*Pigm-1*) and 9311 in Shanghang County in Fujian Province, which has a high incidence of rice blast. We found that 9311(*Pigm-1*) showed resistance to rice blast, while 9311 showed susceptibility to disease (Figure 4b). The number of lesions on the leaves of 9311(*Pigm-1*) was significantly fewer than those on 9311 (Figure 4d).

### 2.6. Genetic Background Analysis of Improved Line 9311(Pigm-1)

**Figure 5:**
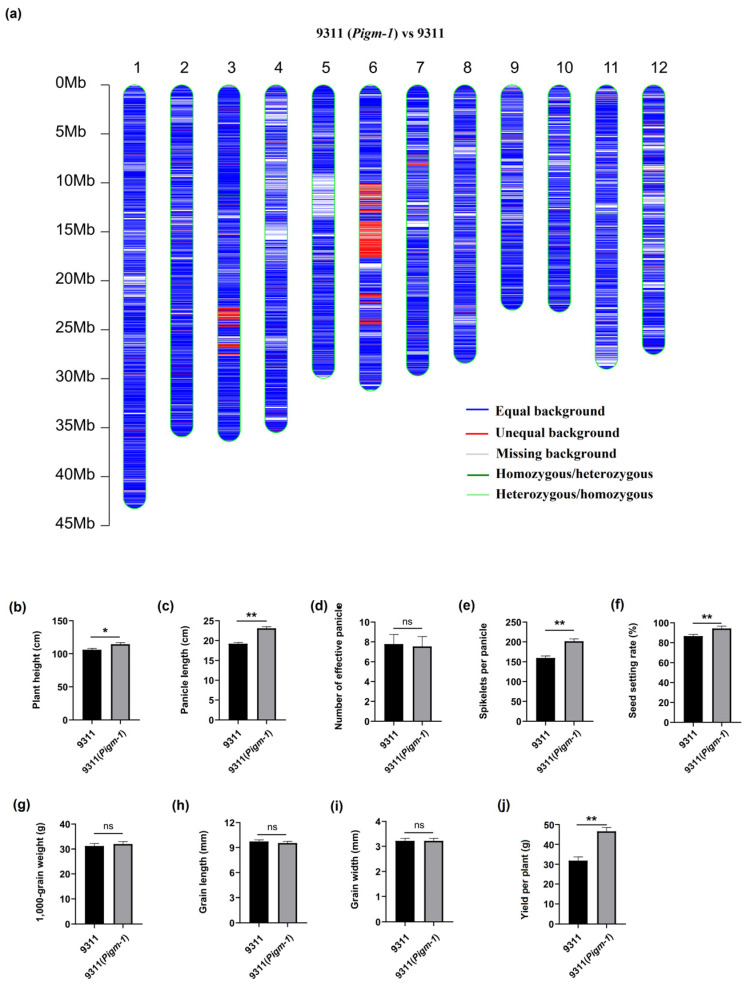
Comparison of the main agronomical traits between 9311 and 9311(*Pigm-1*). (**a**) Genetic background analysis between 9311(*Pigm-1*) and 9311; among them, blue is the same background, red is a different background, white is the missing background of both parents, dark green is the homozygous pair heterozygous background, light green is the heterozygous pair homozygous background. (**b**–**j**) represent the differences between 9311 and 9311(*Pigm-1*) in plant height, panicle length, number of spikelets per panicle, and yield per plant, respectively, according to the results of agronomic traits survey in Supplementary Table S1. Using 9311 as the control, values are expressed as mean ± SE. Asterisks indicate statistical significance determined by Student’s *t*-test. * indicates *p* ≤ 0.05, ** indicates *p* ≤ 0.01, and ns indicates no significant difference.

Whole gene sequencing and analysis of 9311 and 9311(*Pigm-1*) were performed using 4K depth sequencing technology. The results showed that the genetic background of the improved line 9311(*Pigm-1*) was basically restored to the background of wild type 9311 except for the outer regions of chromosomes 3, 6, and 7 (Figure 5a).

### 2.7. Analysis of the Main Agronomic Characteristics of the Improved Line 9311(Pigm-1)

To further analyse the changes in other agronomic traits of the improved line after increasing blast resistance, we investigated the agronomic traits of 9311(*Pigm-1*) and the accepter parent 9311 at maturity. The results showed that compared with 9311, 9311(*Pigm-1*) showed no significant differences in the number of effective panicles, grain length, grain width, and 1000-grain weight (Figure 5b–j and Supplementary Table S4), but showed significant differences in plant height, panicle length, spikelets per panicle, seed-setting rate, and yield per plant (Figure 5b–j and Supplementary Table S4).

### 2.8. Pigm-1-CC Interacts with RAI1

**Figure 6:**
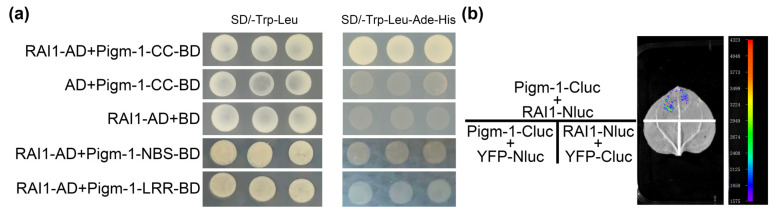
Pigm-1-CC interacts with RAI1. (**a**) The interaction between Pigm-1-CC and RAI1 was detected by Y2H assays. The corresponding vectors were cotransformed into yeast, and the transformed yeast was cultured on the defective medium (SD/-Trp-Leu, SD/-Trp-Leu-Ade-His). Each combination was repeated three times. (**b**) Pigm-1 interacted with RAI1 in the LUC assay. The indicated constructs were transiently coexpressed in 4-week-old *N. benthamiana* leaves. YFP-Nluc and YFP-Cluc empty vectors were used as negative controls.

To confirm that the CC domain may play a crucial role in its nuclear accumulation and binding to transcriptional regulators, we employed a Y2H assay to verify the interaction between the CC domain of Pigm-1 and RAI1 (Figure 6a). To further validate their interaction, we conducted a LUC assay in leaves of *Nicotiana benthamiana*. As shown in Figure 6b, luminescent signals were detected in the leaves when RAI1-Nluc and Pigm-1-Cluc were co-expressed, whereas no signals were detected in the negative controls.

## 3. Discussion

### 3.1. Application Prospect Analysis of the Pigm-1 Gene

The utilisation of resistance genes that confer durable and broad-spectrum resistance against diverse isolates of blast pathogens has been a priority area in rice resistance breeding. Among the various dominant blast resistance genes being considered for breeding resistant varieties, the *PigmR* gene has shown broad-spectrum and durable resistance to blast in different rice production areas around the world [10]. In addition, since varieties derived from resistance gene donors have been cultivated in large areas for many years, resistance genes may decompose due to high selection pressure [21]. Therefore, broad-spectrum rice blast genes have high utilisation value. Meanwhile, *Pigm-1*, the *PigmR* allele, also shows broad-spectrum and durable resistance to blast [21]. In the early stage, we transferred *Pigm-1* into different materials through molecular marker-assisted breeding, and we obtained new materials with different genetic backgrounds for disease resistance. For example, the rice blast resistance of the restorer lines Minghui 86 [21] and R20-1 was improved [22].

In this study, *Pigm-1* was transferred into the susceptible material 9311 to obtain the stable resistant material 9311(*Pigm-1*). Compared with the wild type, 9311(Pigm-1) showed significant differences in plant height, panicle length, spikelets per panicle, seed setting rate, and yield per plant, and the yield traits of 9311(Pigm-1) were better than those of wild-type 9311, possibly because 9311(Pigm-1) contains other fragments in addition to *Pigm-1* (Figure 5a).

Our analysis showed that OsRac1 in the sequenced rice varieties could be divided into four main types, among which ZH11 and 9311 were the most common types. Compared with wild-type OsRac1, OsRac1 in 9311 only differed by one amino acid (Figure 2). Then, we transferred *Pigm-1* into 9311 and obtained the homozygous line 9311(Pigm-1), which showed resistance to disease (Figure 3). Therefore, we speculate that *OsRac1* in 9311 can function normally against disease. *OsRac1* in the other two types of rice varieties are relatively different from that in the wild type (Figure 3). It remains to be further verified whether *OsRac1* in these two types of rice varieties can normally play the function of disease resistance, especially the NIP variety, which differs greatly (Figure 3). Therefore, although *Pigm-1* has strong and broad-spectrum blast resistance, when polymerising *Pigm-1* in molecular marker-assisted breeding in the future, we need to consider whether *OsRac1* has a complete function in the receptor material to improve the efficiency and effect of our molecular breeding.

### 3.2. OsRac1 Is Involved in Pigm-1-Mediated Blast Resistance

Small GTPase Rac/Rop family members act as molecular switches and play a crucial role in various physiological processes in plants [11,12]. Further investigation revealed that OsRac1 acts as a molecular switch by cycling between the inactive form of GDP binding and the active form of GTP binding in cells. Activated GTP-bound Rac/Rop binds to downstream target proteins to control various cellular events [14,15]. The study showed that OsRac1 was verified to directly interact with the NBS domain of several NLR proteins [13]. In this study, the interaction between NBS of Pigm-1 and OsRac1 was verified by the Y2H and LUC methods (Figure 1). To further verify whether OsRac1 directly mediates Pigm-1 resistance, we directly knocked out OsRac1 in the context of Shuangkang 77009 containing *Pigm-1*, and two different knockout lines showed susceptibility (Figure 3). The analysis of agronomic traits showed that compared with wild type Shuangkang 77009, the knockout lines had no significant changes in other agronomic traits except for the effect on blast resistance (Supplementary Table S3). These results suggest that OsRac1 is involved in Pigm-1-mediated blast resistance but does not affect other agronomic traits.

### 3.3. A Model for OsRac1 and RAI1-Mediated Disease Resistance

**Figure 7:**
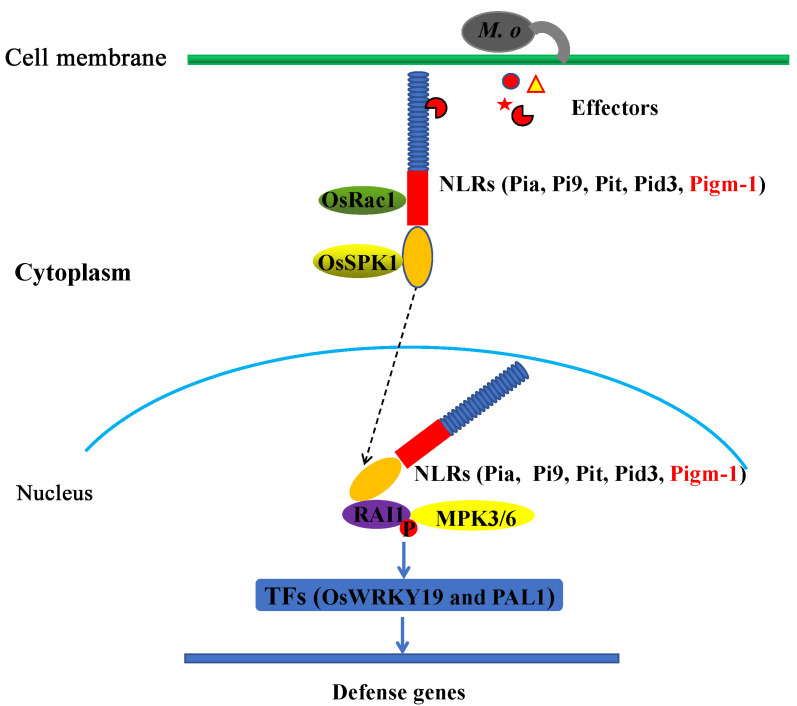
A model of the OsRac1-mediated plant immune response. On or within the membrane, pathogens release effector proteins, and NLR proteins such as Pia, Pit, Pi9, and Pid3 recognise these effector proteins directly or indirectly through the LRR domain. In the cytoplasm, the CC domain of these NLR proteins interacts with OsSPK1, and the NBS domain interacts with OsRac1, stabilising and promoting the incorporation of NLR proteins into the nucleus and binding with RAI1. In the nucleus, OsRac1 binds to OsMAPK3 and phosphorylates the transcription factor RAI1 in the nucleus through the OsMKK4-OsMAPK3/6 cascade reaction to regulate the expression of downstream genes such as PAL1 and OsWRKY19, thus triggering the plant immune response.

It has been found that the small GTPase OsRac1 plays a key role in regulating both PTI and ETI in rice [14,15]. In ETI, based on the results of previous studies, we preliminarily summarised the OsRac1-NLR-mediated immune response model (Figure 7). The GEF protein OsSPK1 was found to bind to the CC domain of Pit and then activate downstream OsRac1 to trigger the immune response. OsSPK1 is also involved in Pia-mediated resistance through interaction with the CC domain of RGA4 [19]. OsRac1 was verified to directly interact with the NBS domain of several NLR proteins, including Pi9, through a Y2H experiment [13]. These findings suggest that OsSPK1, together with OsRac1 and RAI1, may constitute an immune signalling pathway downstream. To verify this hypothesis, we demonstrated through a series of genetic experiments that OsSPK1, OsRac1, and RAI1 are also required to mediate the rice blast resistance of Pi9 and Pid3 [20]. In this study, Pigm-1, which is similar to Pi9, requires OsRac1 to mediate rice blast resistance. Therefore, OsRac1 has been shown to be a common factor downstream of R proteins such as Pia, Pit, Pi9, Pid3, and Pigm-1.

However, how are these NLRs, such as Pia, Pit, Pi9, Pid3, and Pigm-1, transported into the nucleus? Pid3 and Pi9 have previously been reported to bind to RAI1 in the rice nucleus via the CC domain [23]. In fact, such an intracellular protein interaction relationship has also been found in other plant NLRs proteins, such as barley MLA10 and potato Rx1, which also bind corresponding transcription regulatory factors through the CC domain and directly control the protein homeostasis or transcriptional activity of the latter, thus affecting the expression of downstream response genes [24,25,26,27]. We speculate that Pia, Pit, Pi9, Pid3, and Pigm-1 may also adopt a similar mechanism to consolidate and enhance the regulatory role of RAI1 in the downstream immune response. Although it has been jointly suggested that NLR protein transport into the nucleus may be a common mechanism of plant disease resistance, the CC domain may play an important role in its accumulation in the nucleus and its binding to transcriptional regulators.

To verify this hypothesis, we determined that the CC domain of Pigm-1 interacts with RAI1 using a Y2H assay (Figure 6a). To further verify their interaction, we performed a LUC assay in *N*. *benthamiana* leaves. As shown in Figure 6b, luminescent signals were detected in leaves when RAI1-Nluc and Pigm-1-Cluc were coexpressed but not in the negative controls. Therefore, these experiments indicated that the CC domains of Pigm-1 are necessary and sufficient for their physical interaction. However, a nuclear localisation signal has not been detected in many reported CC domains, and it is clear that much research work is still needed to understand the mechanism of NLR proteins’ entry into the nucleus [20,28].

Therefore, based on the above results, in the cytoplasm, we speculate the CC domain of Pigm-1 protein interacts with OsSPK1, and the NBS domain interacts with OsRac1, stabilising and promoting the incorporation of Pigm-1 protein into the nucleus and binding with RAI1 (Figure 7). In the nucleus, RAI1 binds to OsMAPK3 and phosphorylation of RAI1 through the OsMKK4-OsMAPK3/6 cascade promotes its function to regulate the expression of downstream genes such as PAL1 and OsWRKY19, thus triggering the plant immune responses [29,30,31,32] (Figure 7).

## 4. Materials and Methods

### 4.1. Plant Materials

The *indica* rice cultivar Shuangkang 77009 was kept at the Rice Research Institute, Fujian Academy of Agricultural Sciences. The 9311, 9311(*Pigm-1*), Shuangkang 77009, *Rac1 KO-Line1*, and *Rac1 KO-Line2* lines were planted in the summer of 2022 at the Experimental Station in Fujian Province, China. All plants were planted in accordance with standard commercial practices. The plants were grown in the field at a 13.3 cm plant-to-plant distance and a 26.40 cm row-to-row distance. The normal agricultural practices locally recommended for rice production were followed. In addition, plant height, panicle length, effective panicle number, spikelets per panicle, seed-setting rate, and 1000-grain weight were also estimated at the maturity stage for the 9311, 9311(*Pigm-1*), Shuangkang 77009, *Rac1 KO-Line1* and *Rac1 KO-Line2* lines.

The wild type of blast fungus Guy11 was provided by the Plant Protection College of Fujian Agriculture and Forestry University, and Guy11 was used to infect rice leaves as described previously [33].

### 4.2. Experiment on Incubation with Rice Blast Fungus

For spray inoculation, two-week-old rice seedling leaves were sprayed with a spore suspension (1 × 10^5^ spores/mL). The inoculated rice was placed in a moist chamber at 28 °C for 24 h in darkness and then transferred to another moist chamber with a photoperiod of 12 h under light. Each inoculated material (Shuangkang 77009 and the two knockout transgenic lines) was planted in at least three plots, with each plot containing at least nine individual plants.

### 4.3. RNA and DNA Isolation

Total RNA was extracted from the collected samples using TRIzol reagent (Thermo Fisher Scientific, Carlsbad, CA, USA). Complementary DNA (cDNA) was synthesised from 2 μg of total RNA using M-MLV reverse transcriptase (Promega, Madison, WI, USA). DNA was extracted from frozen rice leaves by using the cetyltrimethylammonium bromide method [34] with minor modifications. DNA polymerase chain reaction (PCR) amplification was performed as previously described [35].

### 4.4. Targeted Mutagenesis of OsRac1 with CRISPR/Cas9

The *OsRac1* gene in Shuangkang 77009 was targeted with a gRNA spacer that spanned the two exons of the gene. Highly specific gRNA spacer sequences (Supplementary Table S5) were designed using the CRISPR-plant database and website [36]. Upon transformation, the regenerated plants were analysed for genome editing mutations in the target genes. Individual strains were selected from transgenic CRISPR-edited cell lines for the sequencing of specific mutations in the PCR products [37]. The CRISPR/Cas9 primers used in this study are shown in Supplementary Table S2.

### 4.5. The LUC Assay

*Agrobacterium tumefaciens* strain GV3101 containing the indicated genes was coinjected into *N. benthamiana* leaves and then placed in a growth chamber. Three days after injection, the leaves were sprayed with 1 mM luciferin for CCD imaging, as described previously [38]. For the relative LUC activity assay, leaf discs were collected and incubated with 1 mM luciferin in 96-well plates for 5 min, and luminescence was recorded with the GLOMAX 96 (Promega, Madison, WI, USA) microplate luminometer (vector construction primers are shown in Supplementary Table S2).

### 4.6. Y2H Assay

The Y2H assay was performed as described previously [39]. The indicated genes were incorporated into the pGBKT7 and pGADT7 vectors and then cotransformed into the yeast AH109 strain (Clontech, Palo Alto, CA, USA) and grown on SD/-Trp/-Leu plates for three days. Positive clones were selected and diluted with 50 µL of sterile water, and then 10 µL of suspension was pipetted onto SD selection medium for incubation (vector construction primers are shown in Supplementary Table S2).

### 4.7. Bioinformatics Analysis

Multiple sequences were analysed and compared according to the following database: http://multalin.toulouse.inra.fr/multalin/multalin.html (accessed on 1 November 2022).

### 4.8. Statistical Analysis

Differences among traits were identified through one-way Analysis of Variance (ANOVA), with the results presented as mean ± standard error (or standard deviation, noting that the n value is indicated in the figure legends). The experimental data were then analysed utilising the SPSS 23.0 and OriginPro 9.0 plus software (accessible at https://www.originlab.com).

## 5. Conclusions

Our study underscores the vital role of NLR proteins in rice’s defence against *Magnaporthe oryzae*-induced blast. Despite their importance, the intricate mechanism by which these R proteins regulate downstream signalling remains largely unexplored due to a lack of knowledge on their direct targets. By cloning *Pigm-1*, a novel allele of *PigmR* from Shuangkang 77009, we have shed light on the signalling cascade initiated by NLRs. We identified OsRac1, a GTPase, as a key signalling molecule involved in Pigm-1-mediated blast resistance, suggesting that OsRac1 is a universal downstream effector for rice NLRs. Furthermore, RAI1, a transcriptional activator, emerged as a crucial Pigm-1 partner, with Pigm-1’s NBS and CC domains facilitating the activation of OsRac1 and RAI1, respectively, leading to cell death. Importantly, our research demonstrates that molecular marker-assisted selection can simultaneously enhance resistance and yield in the two-line restorer 9311(*Pigm-1*). Overall, this study provides invaluable insights into NLR signalling and serves as a reference for breeding rice blast resistance genes, especially *Pigm-1*.

## Data Availability

Data is contained within the article.

## Supplementary Materials

The following supporting information can be downloaded at: https://www.mdpi.com/article/10.3390/plants14020217/s1, Figure S1: Amino acid sequence analysis and comparison between Pigm-1 and Pit, Pia, Pid3, and Pi9. The green underlined letters indicate the predicted NBS domains of each of the four NLR proteins; Figure S2: Construction of new rice blast resistant plants with 9311 as recipient; Table S1: Amino acid sequence analysis of *OsRac1* in different rice varieties; Table S2: Primer sequences used for carrier construction and genotyping CRISPR-edited mutants; Table S3: Comparison of the main agronomical traits between Shuangkang 77009 and two knockout transgenic lines; Table S4: Comparison of the main agronomical traits between 9311 and 9311(*Pigm-1*); Table S5: Mutation site of two target mutant lines.
