# Supplementary material for: Importance of OsRac1 in Signalling of Pigm-1 Mediated Resistance to Rice Blast Disease
Source: Plants (Basel). 2025 Jan 14;14(2):217. doi: 10.3390/plants14020217 (PMC11769553; doi:10.3390/plants14020217)
Supplement: Supplementary file 1 [file plants-14-00217-s001.zip › plants-3274473-supplementary.pdf]

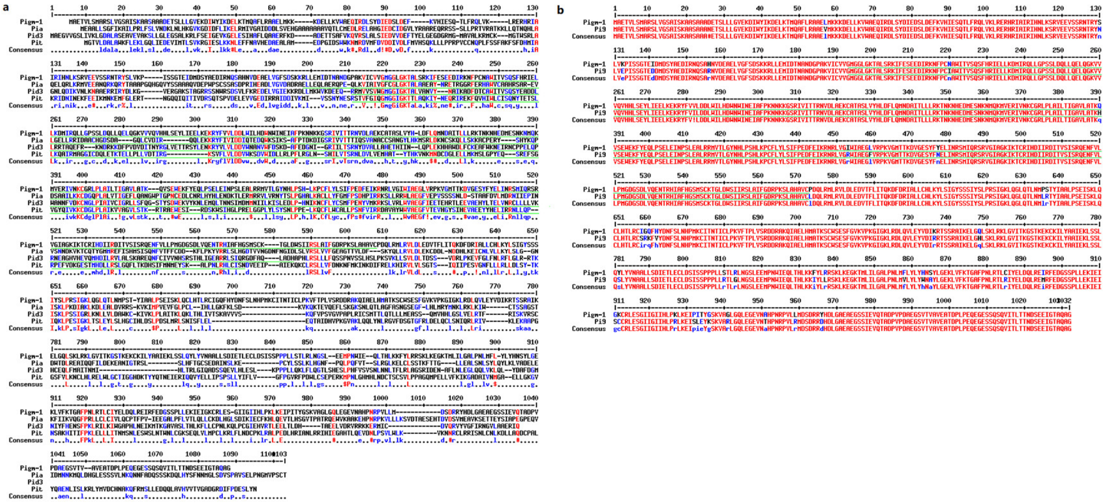

Supplementary Figure 1. Amino acid sequence analysis and comparison between Pigm-1 and Pit, Pia, Pid3, and Pi9. The green underlined letters indicate the predicted NBS domains of each of the four NLR proteins.

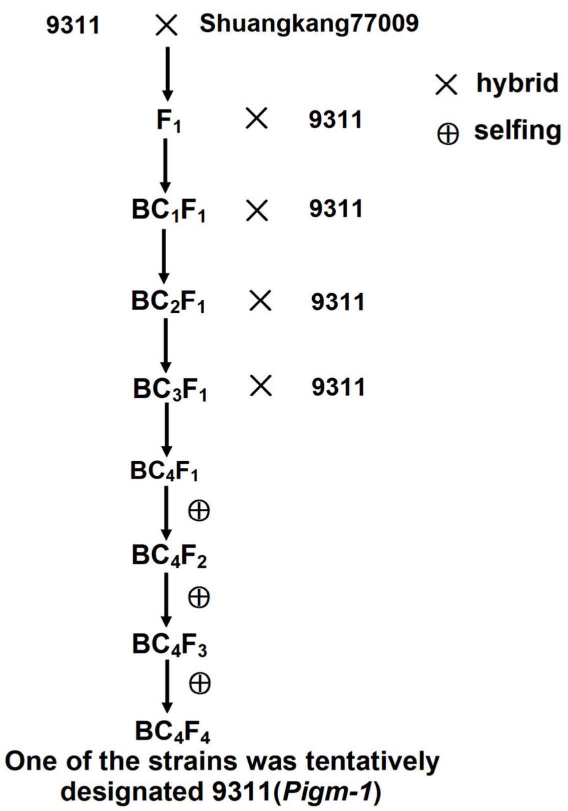

Supplementary Figure 2. Construction of new rice blast resistant plants with 9311 as recipient.

Supplementary Table 1. Amino acid sequence analysis of *OsRac1* in different rice varieties.

Material name	Amino acid sequence	Change site
ZH11, CG14, KY131, LJ, NamRoo, Kosh, DHX2	MSSAAAATRFIKCVTVGDGAVGKTCMLICYTCNKFPTDYIPTVFDNFSANVS<br>VDGSVVNLGLWDTAGQEDYSRLRPLSYRGADVFILSFSLISRASYENVQKKW<br>MPELRRFAPGVPVVLVGTKLDLREDRAYLADHPASSIITTEQGEELRKLIGAV<br>AYIECSSKTQRNIKAVFDTAIKVVLQPPRHKDVTRKKLQSSSNRPVRRYFCGS<br>ACFA	No change
9311, 02428, IR64, CN1, D62, R498, Lemont FH838, FS32, G46, G8, G630, II32, J4155, DG, Y3551, R527, S548, Tumba, WSSM, Y58S, YX1	MSSAAAATRFIKCVTVGDGAVGKTCMLICYTCNKFPTDYIPTVFDNFSANVS<br>VDGSVVNLGLWDTAGQEDYSRLRPLSYRGADVFILSFSLISRASYENVQKKW<br>MPELRRFAPGVPVVLVGTKLDLREDRAYLADHPASSIITMEQGEELRKLIGAV<br>AYIECSSKTQRNIKAVFDTAIKVVLQPPRHKDVTRKKLQSSSNRPVRRYFCGS<br>ACFA	T144 to M144
Basmati1, TM, N22	MSSAAAATRFIKCVTVGDGAVGKTCMLICYTCNKFPTDYIPTVFDNFSANVS<br>VDGSVVNLGLWDTAGQEDYSRLRPLSYRGADVFILSFSLISRASYENVQKKW<br>MPELRRFAPGVPVVLVGTKLDLREDRAYLADHPTSSIITMEQGEELRKLIGAV<br>AYIECSSKTQRNIKAVFDTAIKVVLQPPRHKDVTRKKLQSSSNRPVRRYFCGS<br>ACFA	T144 to M144 and A138 to T138
NIP	MSGATKFIKCVTVGDGAVGKTCMLICYTSNKFPTDYIPTVFDNFSANVSVDG<br>NIVNLGLWDTAGQEDYSRLRPLSYRGADIFVLAFSLISRASYENVLKKWMPE<br>LRRFAPNVPIVLVGTKLDLRDHRSYLADHPAASAITTAQGEELRKQIGAAAYI<br>ECSSKTQQNIKAVFDTAIKVVLQPPRRRGETTMARKKTRRSTGCSLKNLMCG<br>SACVV	Multisite change
Shuangkang 77009	MSSAAAATRFIKCVTVGDGAVGKTCMLICYTCNKFPTDYIPTVFDNFSANVS<br>VDGSVVNLGLWDTAGQEDYSRLRPLSYRGADVFILSFSLISRASYENVQKKW<br>MPELRRFAPGVPVVLVGTKLDLREDRAYLADHPASSIITTEQGEELRKLIGAVA<br>YIECSSKTQRNIKAVFDTAIKVVLQPPRHKDVTRKKLQSSSNRPVRRYFCGSA<br>CFA	No change

Supplementary Table 2. Primer sequences used for carrier construction and genotyping CRISPR-edited mutants.

Name	sequence 5’→3’	Purpose
Osrac1-F	GCCGCTAATCCCTTCCTCTC	Screening of lines
Osrac1-R	TGCATCAGAATGACAAGGGG	Screening of lines
OsRac1-AD-F	GTACCAGATTACGCTCATATGATGAGCTCGGCGGCGGCGGC	Interaction verification
OsRac1-AD-R	ATGCCCACCCGGGTGGAATTCCTACGCGAAACAAGCGCTTC	Interaction verification
OsRac1-Nluc-F	ACGGGGGACGAGCTCGGTACCATGAGCTCGGCGGCGGCGGC	Interaction verification
OsRac1-Nluc-R	CGCGTACGAGATCTGGTCGACCTACGCGAAACAAGCGCTTC	Interaction verification
Pigm-1-Cluc-F	TACGCGTCCCGGGGCGGTACCATGGCGGAGACGGTGCTGAG	Interaction verification
Pigm-1-Cluc-R	ACGAAAGCTCTGCAGGTCGACTCAGCCAGCTTGAGCTGTGC	Interaction verification
Pigm-1-NBS-BD-F	TCAGAGGAGGACCTGCATATGCAAGGAGAAGAGGTACTTTGTT	Interaction verification
Pigm-1-NBS-BD-R	TCGACGGATCCCCGGGAATTCATCCTCAGGAAAGATACTTAGATACAAAA	Interaction verification
OsRac1-CDs-F	ATGAGCTCGGCGGCGGCGGC	Amplified CDs
OsRac1-CDs-R	CTACGCGAAACAAGCGCTTC	Amplified CDs
Ral1-CDs-F	ATGGAGCTTGACGAGGAGTC	Amplified CDs
Ral1-CDs-R	CTACAGACACCTTCCGCCAT	Amplified CDs
Pigm-1-CC-BD-F	TCAGAGGAGGACCTGCATATGCACCATGGCGGAGACGGTGCTGAG	Interaction verification
Pigm-1-CC-BD-R	TCGACGGATCCCCGGGAATTCTCATTTGGCCGGACCATCATTAGC	Interaction verification
RAI1-AD-F	GTACCAGATTACGCTCATATGATGGAGCTTGACGAGGAGTC	Interaction verification
RAI1-AD-R	ATGCCCACCCGGGTGGAATTCCTACAGACACCTTCCGCCAT	Interaction verification
RAI1-Nluc-F	ACGGGGGACGAGCTCGGTACCATGGAGCTTGACGAGGAGTC	Interaction verification
RAI1-Nluc-R	CGCGTACGAGATCTGGTCGACCTACAGACACCTTCCGCCAT	Interaction verification

Supplementary Table 3. Comparison of the main agronomical traits between Shuangkang 77009 and two knockout transgenic lines.

Trait	Shuangkang 77009	*Rac1 KO-Line1*	*Rac1 KO-Line2*
Plant height (cm)	113.22 ± 2.82	115.12 ± 2.89	115.22 ± 2.76
Panicle length (cm)	21.84 ± 1.41	21.88 ± 1.37	22.10 ± 1.38
Number of effective panicles	21.82 ± 2.26	22.45 ± 2.34	22.66 ± 2.18
Spikelets per panicle	185.24 ±4.88	186.76 ±5.01	188.76 ±4.98
Seed setting rate (%)	85.74 ± 2.16	84.22 ± 2.46	83.14 ± 2.32
1,000-grain weight (g)	25.32± 0.78	25.01± 0.81	24.82± 0.63
Grain length (mm)	8.81 ± 0.23	8.84 ± 0.26	8.85 ± 0.24
Grain width (mm)	2.90 ± 0.07	2.91 ± 0.08	2.94 ± 0.09

Note: The data was derived from the trial that was performed at the Fuzhou experimental station in October 2022. *P<0.05 and **P<0.01 for the differences between Shuangkang 77009 and two knockout transgenic lines.

Supplementary Table 4. Comparison of the main agronomical traits between 9311 and 9311(*Pigm-1*)

Trait	9311	9311(*Pigm-1*)
Plant height (cm)	106.2 ± 2.12	114.4 ± 2.34*
Panicle length (cm)	19.2 ± 0.31	23.1 ± 0.34**
Number of effective panicles	7.86 ± 1.18	7.54 ± 1.04
Spikelets per panicle	159.7 ± 4.86	201.6 ± 6.22**
Seed setting rate (%)	86.85 ± 1.66	94.26 ± 2.26**
1,000-grain weight (g)	31.16± 0.82	31.92± 0.91
Grain length (mm)	9.72 ± 0.21	9.54 ± 0.19
Grain width (mm)	3.22 ± 0.10	3.22 ± 0.10
Yield per plant (g)	31.62± 1.78	45.73 ± 2.02**

* and ** indicate the significance levels of the differences between 9311 and 9311(*Pigm-1*) were revealed by the *t*-test at $P<0.05$ and $P<0.01$, respectively. The data was derived from the trial that was performed at the Fuzhou experimental station in October 2022.

Supplementary Table 5. Mutation site of two target mutant lines.

Line	Target sequence	Mutation site
Rac1 KO-Line1	CCAGAGGCCGAGG TTGACGACGC	CCACCTGCAGTGTCCCAGAG-------GTTGACGACGCTCCC (7 bp deletion)
Rac1 KO-Line2	CCAGAGGCCGAGG TTGACGACGC	CCACCTGCAGTGTCCCAGAGAAGCCGAGGTTGACGA (2 bp insertion)
